# Supplementary material for: Oncogenic Linear Collagen VI of Invasive Breast Cancer Is Induced by CCL5
Source: J Clin Med. 2020 Apr 2;9(4):991. doi: 10.3390/jcm9040991 (PMC7230614; doi:10.3390/jcm9040991)
Supplement: Supplementary file 1 [file jcm-09-00991-s001.zip › jcm-740450-SI.docx]

H2: Supplementary Materials

Supplemental methods:

Mass spectrometry.

Proteolysis by FASP: After reduction and alkylation using DTT and IAA, the proteins were centrifuged on a 30 kDa cutoff filter device (Sartorius), washed three times with UA buffer (8 M urea in 0.1 M Tris/HCl pH 8.5) and twice with 50 mM ammoniumbicarbonate. The proteins were digested for 2 hours at room temperature using 0.5 µg Lys-C (Wako Chemicals, Neuss, Germany) and for 16 hours at 37°C using 1 µg trypsin (Promega, Mannheim, Germany). After centrifugation (10 min at 14 000 g) the eluted peptides were acidified with 0.5% TFA and stored at -20°C.

LC-MS/MS: LC-MS/MS was performed on a Q-Exactive HF mass spectrometer (Thermo Scientific) online coupled to an Ultimate 3000 nano-RSLC (Dionex). Tryptic peptides were automatically loaded on a C18 trap column (300 µm inner diameter (ID) × 5 mm, Acclaim PepMap100 C18, 5 µm, 100 Å, LC Packings) at 30µl/min flow rate prior to C18 reversed phase chromatography on the analytical column (nanoEase MZ HSS T3 Column, 100Å, 1.8 µm, 75 µm x 250 mm, Waters) at 250nl/min flow rate in a 95 minutes non-linear acetonitrile gradient from 3 to 40% in 0.1% formic acid. Profile precursor spectra from 300 to 1500 m/z were recorded at 60000 resolution with an automatic gain control (AGC) target of 3e6 and a maximum injection time of 50 ms. TOP10 fragment spectra of charges 2 to 7 were recorded at 15000 resolution with an AGC target of 1e5, a maximum injection time of 50 ms, an isolation window of 1.6 m/z, a normalized collision energy of 28 and a dynamic exclusion of 30 seconds.

Quantitative data analysis: Generated raw files were analyzed using Progenesis QI for proteomics (version 4.0, Nonlinear Dynamics, part of Waters) for label-free quantification as described previously (31, 32). Features of charges 2-7 were used and all MSMS spectra were exported as mgf file. Peptide search was performed using Mascot search engine (version 2.6.2) against the Swissprot human protein database (20237 sequences, 11451954 residues). Search settings were: 10 ppm precursor tolerance, 0.02 Da fragment tolerance, one missed cleavage allowed. Carbamidomethyl on cysteine was set as fixed modification, deamidation of glutamine and asparagine allowed as variable modification, as well as oxidation of methionine and Met-loss combined with acetylation at the N-terminus of the protein. Applying the percolator algorithm (34), resulted in a peptide false discovery rate (FDR) of < 1 %. Search results were reimported in the Progenesis QI software. Proteins were quantified by summing up the abundances of all unique peptides per protein. Resulting normalized protein abundances were used for calculation of fold-changes and statistical values were exported from the Progenesis QI software.

Supplementary figure 1:


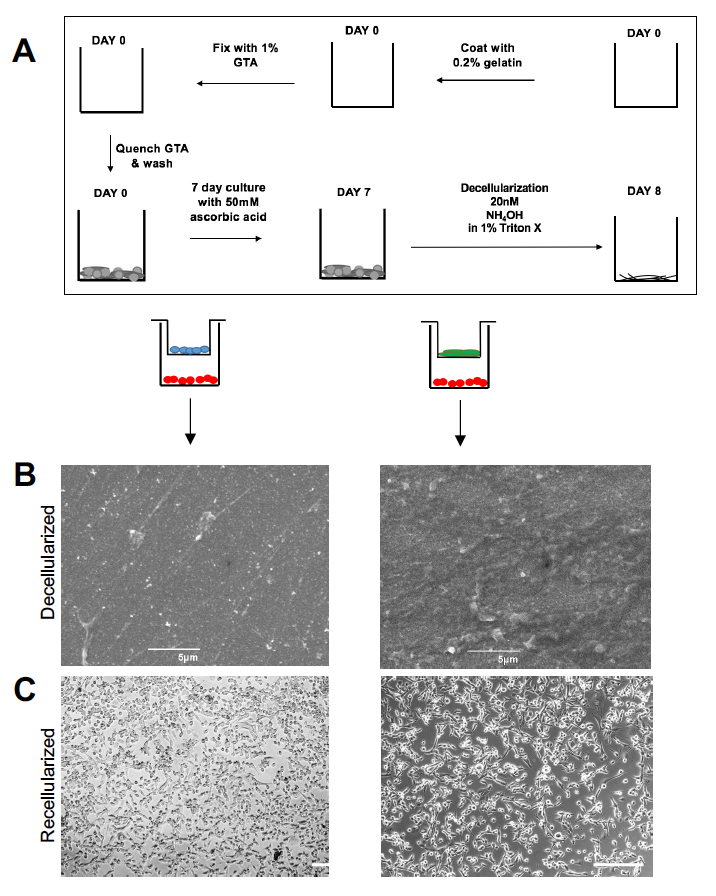


**Supplementary figure 1:** Schematic showing workflow for matrix deposition. (B) SEM images of decellularized matrix deposited by cancer cells under paracrine influence of ASCs (left, blue) and fibroblasts (right, green). (C) Reseeded cells on matrices shown in (B). Scale bar 50μm.

Supplementary figure 2:


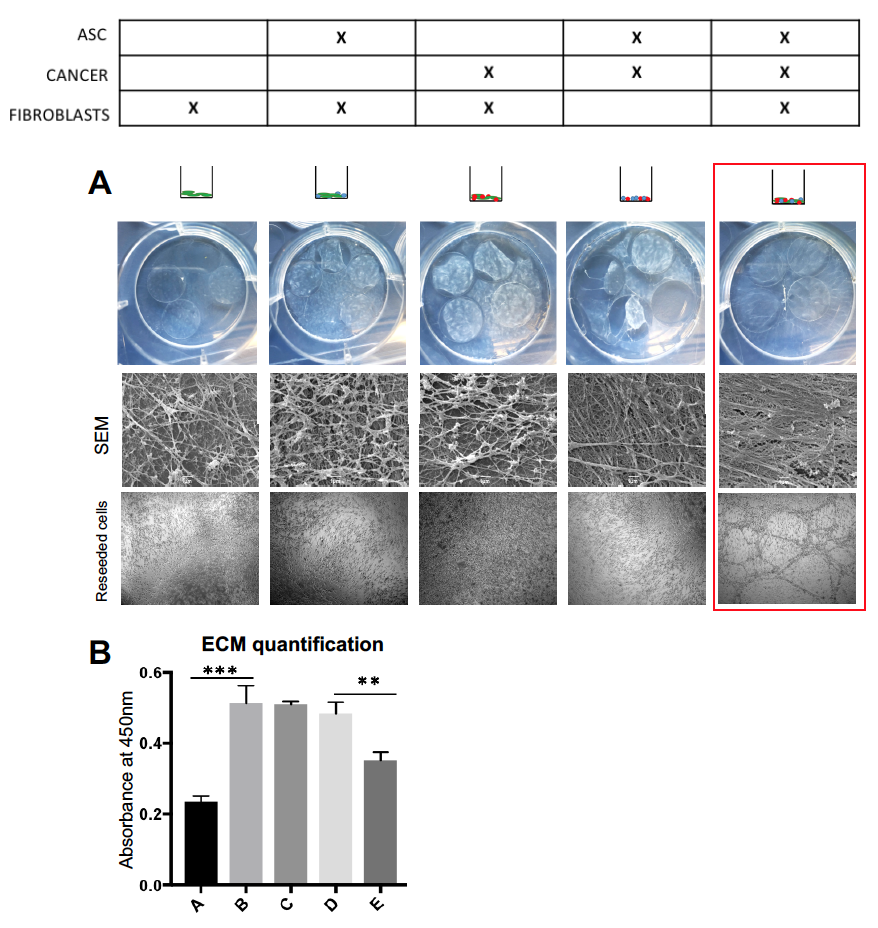


**Supplementary figure 2:** (A) Panel of gross images and SEM at 5000x and 10000x for the initial groups, screened for linear matrices. Red box indicates cancer juxtacrine group comprised of ASCs, MDA-MB-231, and fibroblasts. (B) ECM quantification of all 5 groups. Error bars represent SD. (***p* <0.01, ****p* <0.001).

Supplementary figure 3:


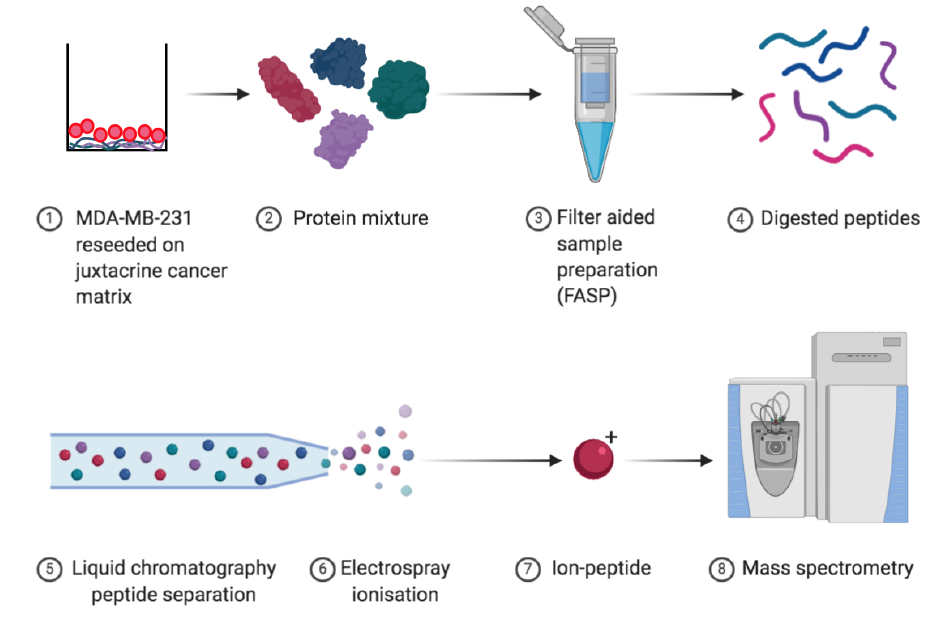


**Supplementary figure 3:** (A) Schematic created with biorender.com, showing mass spectroscopy sample specification and workflow.
